# HIV testing and prevalence in fishing communities in rural Uganda: a cross-sectional study of 3197 individuals within SchistoTrack

**DOI:** 10.1136/bmjopen-2025-108718

**Published:** 2026-02-04

**Authors:** Hanh Lan Bui, Lauren Wilburn, Salim W Nsimbe, Betty Nabatte, Geoffrey W Oromcan, Raymond Mujuni, Juma Nabonge, Narcis B Kabatereine, Adrian Smith, Goylette Chami

**Affiliations:** 1 Big Data Institute, Nuffield Department of Population Health, University of Oxford, Oxford, UK; 2 Uganda Institute of Allied Health and Management Sciences, Kampala, Uganda; 3 Division of Vector Borne Diseases and Neglected Tropical Diseases, Uganda Ministry of Health, Kampala, Uganda; 4 Pakwach District Local Government, Republic of Uganda Ministry of Health, Kampala, Central Region, Uganda; 5 Buliisa District Local Government, Republic of Uganda Ministry of Health, Butiaba, Central Region, Uganda; 6 Mayuge District Local Government, Republic of Uganda Ministry of Health, Mayuge, Central Region, Uganda; 7 Nuffield Department of Population Helath, University of Oxford, Oxford, UK

**Keywords:** HIV & AIDS, Epidemiology, Public health, Community Participation, Infection control

## Abstract

**Abstract:**

**Objectives:**

To compare HIV testing coverage, prevalence and care cascade engagement between fisherfolk and the general population, and to assess the relevance of individual and community-level definitions of fisherfolk in understanding variation in HIV status and testing.

**Design:**

Primary data collection and cross-sectional analysis in 1 year of the SchistoTrack community-based cohort.

**Setting:**

52 shoreline villages in Pakwach, Buliisa and Mayuge districts in rural Uganda.

**Participants:**

A total of 3197 individuals aged 5–92 years were tested for HIV in 2024. A subset of 124 HIV-positive participants had viral load measured in 2025. Statistical analyses focused on 1931 adults aged 15 years and older.

**Primary and secondary outcome measures:**

The primary outcomes were lifetime HIV testing, testing in the past 12 months and current HIV status. Secondary measures included self-reported care cascade outcomes and viral load suppression.

**Results:**

Overall, 6.94% (134/1931) of adult participants aged 15 years and older were with HIV (people with HIV (PWH)), of whom 22.39% (30/134) were newly diagnosed. 6% (25/415) of adults reporting fishing activities were HIV-positive. Of those, 80% (20/25) were status-aware, 76% (19/25) were on antiretroviral therapy, and 100% (8/8) of those who knew their viral load reported viral suppression. No significant differences in care cascade engagement were found between PWH reporting fishing activities and the general population. Measured viral suppression was 70.59% (72/102) among PWH with no significant differences by fishing activities. Fishing activities were significantly associated with higher odds of ever testing for HIV (OR 1.76 (95% CI 1.22 to 2.54)), but not with testing in the past 12 months or HIV status. No consistent district-level differences were observed.

**Conclusions:**

Individuals reporting fishing activities had higher lifetime testing and comparable HIV prevalence and care cascade engagement to the general population. Gaps remain in recent testing, status awareness and viral suppression for fisherfolk.

**STRENGTHS AND LIMITATIONS OF THIS STUDY**

- Several individual and community-level definitions of fisherfolk were incorporated to assess their relevance for HIV-related outcomes.
- Multiple modelling approaches were used, including unadjusted, minimally adjusted and strict Bayesian information criterion-based variable-selection models, to ensure the robustness of associations.
- Care cascade estimates were based on prevalent rather than follow-up data and relied solely on self-reports, potentially introducing recall and social desirability biases.
- Viral load measurements were available only for a subset of HIV-positive participants and were collected 12 months after self-reported care cascade data.

## Introduction

 In 2023, sub-Saharan Africa (SSA) accounted for nearly 65% of the global HIV burden, with 39.9 million people with HIV (PWH) worldwide.^1^ The HIV epidemic in SSA is more generalised than in other regions of the world, although key populations, such as men who have sex with men, people who inject drugs and sex workers, now account for 51% of new infections.^2 3^ The HIV response in SSA is guided by the Joint United Nations Programme on HIV and AIDS (UNAIDS) 95–95–95 targets, which aim to end the AIDS epidemic by 2030.^4^ WHO policy advises that all individuals diagnosed with HIV should have immediate access to antiretroviral therapy (ART).^5^ Increasing status awareness among PWH through timely testing is the entry point to accessing the HIV care cascade.^6^

Countries in SSA have identified priority populations in need of targeted efforts for HIV testing and linkage to care.^7^ In many SSA countries, including Uganda, an important priority population is fisherfolk—residents of fishing communities—comprising fishermen, fishmongers and others involved in the fishing trade.^8^ HIV prevalence in fisherfolk is heterogeneous across SSA and is estimated at 15–40% in Uganda (based on studies conducted between 2009 and 2016), primarily assessed in fishing communities around Lake Victoria.^7^,^9^ The most recent estimate is 17.5% around Lakes Edward and George in 2016^10^. HIV testing coverage among fisherfolk also varies widely across studies and contexts; Opio *et al* reported 54% in 2013, while the Rakai Community Cohort Study recorded an increase from 68% in 2011–2012 to 96% in 2016–2017 following provision of HIV testing and counselling.^8 11 12^

Fisherfolk in Uganda and elsewhere are defined as a priority population for HIV services due to multiple structural, behavioural and sociodemographic barriers to consistent engagement with care.^810 13^,^16^ High levels of mobility, driven by seasonal fishing cycles and movement between landing sites, are a key factor contributing to their heightened vulnerability to HIV.^8 10 15^ Mobility is gendered: men typically migrate seasonally for fishing, often spending months moving between fishing villages, while women involved in the fish trade tend to move less frequently.^16^ Fishing communities are often located in remote, under-resourced areas, with long distances to health facilities, inadequate transportation, limited access to healthcare and poor healthcare infrastructure.^8 13 15^ These structural barriers impede access to HIV testing and care, while risk behaviours such as transactional and commercial sex and alcohol use add to HIV acquisition risk among fisherfolk.^8 14^

While community-based outreach initiatives, including HIV self-testing and mobile outreach, have sought to mitigate structural barriers, data to evidence their effectiveness remain limited.^10 17 18^ There is therefore a need to establish care cascades among fisherfolk and to contrast these with those of the general populations. Further, HIV interventions and research with fishing communities have generally been conducted at the community level, sampling from landing sites where the entire village or community is treated as one unit.^10^,^1219 20^ However, it is unclear whether such ecological associations with higher HIV prevalence or lower testing among fisherfolk also hold at the individual level, underscoring the need for more precise definitions to effectively target this priority group in HIV interventions. Differences in sampling across studies make it challenging to assess the true prevalence of testing and PWH among the priority population of fisherfolk.^10^,^1219 20^

Here, we examined HIV prevalence, testing coverage and the care cascade in fishing communities in rural Eastern and Western Uganda. A total of 3197 individuals aged 5–92 were screened for HIV using the Uganda Ministry of Health national testing algorithm within the community-based cohort, SchistoTrack. The primary purpose of the SchistoTrack cohort is to look at liver and spleen disease progression within communities that have a high burden of schistosomiasis and coendemic infections such as HIV. The overall aims of this study were to compare HIV testing coverage, prevalence and care cascade engagement between fisherfolk and the general population, and to assess the relevance of individual- and community-level definitions of fisherfolk in understanding variation in HIV status and testing.

## Methods

### Study design and context

Collection of primary data for HIV diagnoses, history of infection and diagnoses, and ART administration was added to the community-based SchistoTrack cohort in 2024 to conduct a nested cross-sectional study on HIV patterns within fishing communities. SchistoTrack was established in January 2022 and examined participants aged 5 years and older from 52 shoreline villages in Pakwach, Buliisa and Mayuge districts.^21^ All residents aged 5 years and older were eligible. Households were randomly selected, and recruitment was carried out through community mobilisation with village health teams, followed by household visits during which one child and one adult per household were enrolled. More details about the cohort and sampling can be found in Puthur *et al*.^21^ HIV testing was introduced following observation of a correlation between self-reported HIV status and periportal fibrosis.^22^ From 15 January to 12 February 2024, study participants were tested for HIV using rapid diagnostic tests following the Uganda national algorithm and completed a structured HIV history survey with a district HIV counsellor. Viral load was subsequently collected for a subset of HIV-positive participants 12 months later.

### Outcomes

The primary outcomes of the study were lifetime HIV testing (ever testing) and current HIV infection status (both binary). To compare the determinants of testing recency with those of ever testing, we also examined HIV testing in the past 12 months among participants who self-reported being HIV-negative. HIV status was determined according to the Uganda Ministry of Health national testing algorithm based on three tests: Determine HIV-1/2 (Abbott) as a first-pass screening test, HIV 1/2 STAT-PAK Assay (Chembio Diagnostics) as a confirmatory test and Bioline HIV 1/2 3.0 (Abbott) as a tie-breaker where the first two tests disagreed (online supplemental figure S2).^9^

**Figure 1:**
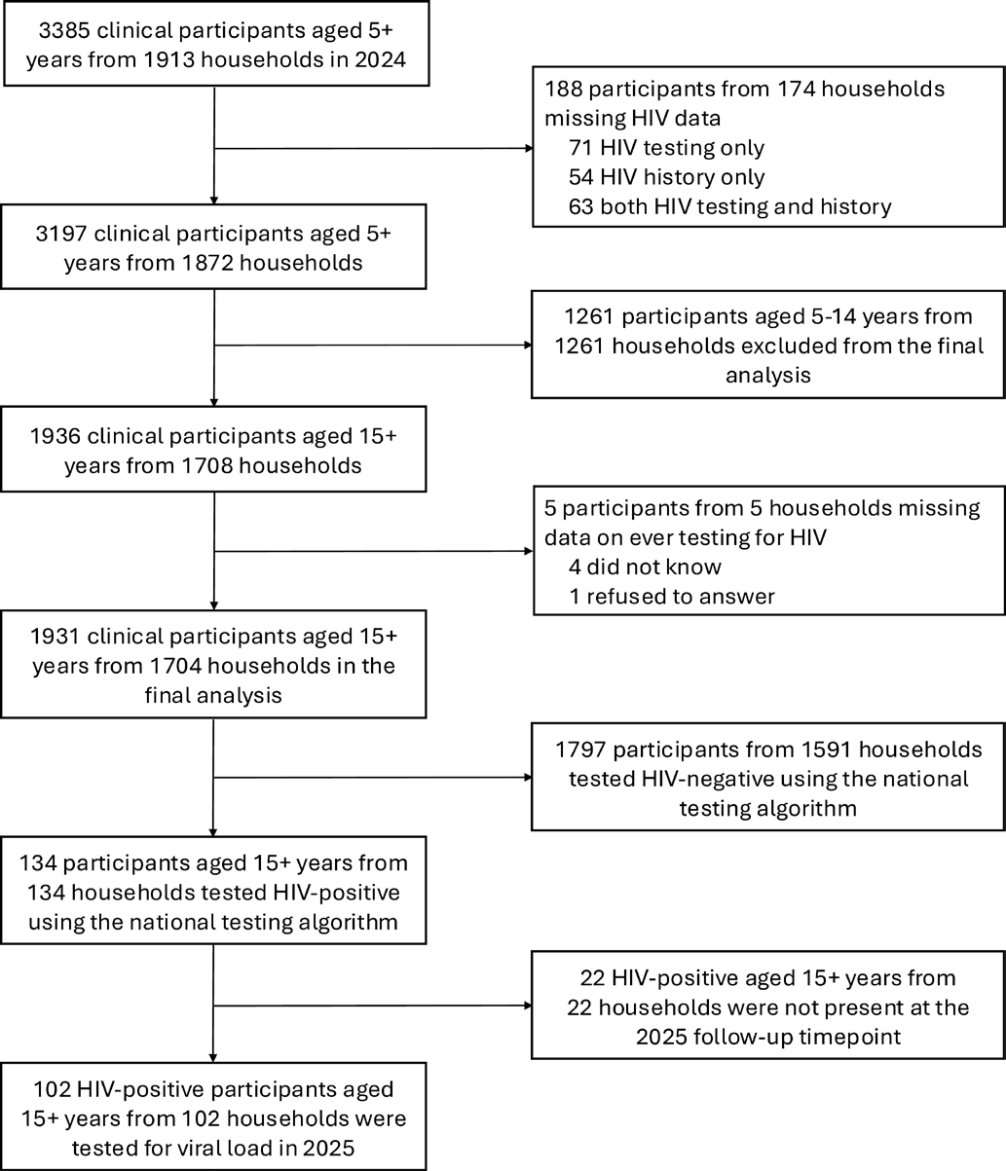
Participant flow diagram.

The care cascade measures among PWH were self-reported in the 2024 HIV history survey and included HIV status awareness, linkage to care (defined as seeing a healthcare provider for the first time within 30 days of diagnosis), ART initiation (ever taking ART to treat HIV), current ART use, retention in care (defined as receiving HIV care from a healthcare provider in the past 12 months) and self-reported HIV viral load suppression (here defined as self-reported viral load of <1000 copies/mL) (online supplemental table S2). Additionally, PWH were asked to report the HIV clinic where they accessed ART; for newly diagnosed individuals, the clinic associated with their newly issued ART number was recorded. Participants who did not know or refused to report their lifetime testing history were excluded from all analyses (figure 1). Those who did not know or refused to answer other self-reported outcomes were excluded only from analyses involving those specific outcomes.

Samples for viral load quantification were collected for a subset of PWH who attended the follow-up event in January 2025 (124 out of 163 eligible by HIV positivity on Determine). Viral load was collected and analysed using dried blood spots (DBS) (m2000sp), and viral suppression was defined as a binary indicator of a viral load result of <1000 copies/mL, in line with WHO criteria.^5 23^ Detailed information on HIV data collection is provided in online supplemental methods.

### Fisherfolk definitions

The household survey was conducted at recruitment, with participants enrolled between 2022 and 2024. It was administered by local surveyors fluent in Lusoga, Lugungu, Alur and English. The survey collected demographic, socioeconomic and health behavioural data, along with information on water-contact activities, household locations and government health centre locations. Six definitions of fisherfolk were tested: three were individual-level and three were community-level. Two individual-level binary classifications were constructed based on participant reports of fishing activities (catching fish) and fishmongering activities (selling fish) among 11 water-contact activities, as detailed elsewhere.^24^ We also examined a categorical occupation variable at the individual level, with categories including fisherman, fishmonger, subsistence farmer and rice farmer, using none or other as the reference group. Community-level definitions were based on household distance to the nearest water site (continuous), the presence of a landing site (binary) and the presence of a beach in the village (binary).

This analysis also included individual and household-level covariates covering sociodemographic, health behavioural and spatial factors, as listed in online supplemental methods. Here, we defined adult study participants as aged 15 years or older. Detailed definitions of the covariates are provided elsewhere.^21^

### Statistical analysis

Data were collected through digital surveys created using Open Data Kit Collect (V.2022.4.2, 2023.2.4 and 2024.3), with data entered using Android tablets (Android V.9.0 Pie software). Analyses were performed using R V.4.2.1.^25^ Geographic coordinates of reported HIV clinics were geocoded using the Google Maps Geocoding Application Programming Interface and mapped to Uganda district shapefiles.^26 27^ Geodesic distances were calculated between households and HIV clinics.^28^ Descriptive analyses of HIV testing and status trends were conducted by constructing kernel density plots over age, stratified by gender, for all participants in the cohort (3197 participants aged 5–92 years). Each density curve represented a smoothed estimate of the underlying age distribution for the corresponding outcome, calculated using a Gaussian kernel with the default bandwidth (Silverman’s rule of thumb).^25^

Only participants (1931 participants from 1704 households) aged 15–92 years (referred to as adults for reporting purposes, even though this age group includes older adolescents) were included in the subsequent analyses described in order to be comparable with Uganda Population-based HIV Impact Assessment (UPHIA) national statistics.^29^ Study participants younger than 15 years were excluded from further analyses. Since the age distribution of the SchistoTrack population sample is bimodal, study HIV prevalence estimates were directly age-standardised to the UPHIA 2020–2021 population age structure for comparison with national HIV prevalence estimates. For this, participants were grouped into 10-year age strata, with those aged 65 and above grouped together.

To better understand the social, behavioural and spatial factors associated with HIV testing and HIV infection status, we constructed adjusted logistic regression models. The models were specified through backwards stepwise model selection using the lowest Bayesian information criterion (BIC).^25^ The candidate covariate list was guided by determinants found relevant in published literature and included the six definitions of fisherfolk and other covariates detailed above.^8 30 31^ Age, gender and district were forced into the models. Additionally, fisherfolk definitions were assessed using unadjusted and minimally adjusted logistic regression models (controlling for age, gender and district) to identify the most relevant definition based on associations with ever testing, testing in the past 12 months and HIV status. One participant was missing information on tribal membership, and another on alcohol and smoking; these two participants were excluded during stepwise model selection but included in the final models, as those variables were not retained. 95% CIs for ORs in BIC-based models were derived from standard errors clustered at the village level, to account for the sampling design of households nested within villages.^32^ Floating absolute risks were also calculated for the district variable to assess the chosen reference category.^33^ Intraclass correlation coefficients for empty multilevel models at the village level were calculated to assess variations within and between clusters for the study outcomes.^34^ Variance inflation factors were computed to assess multicollinearity.^35^ Although the main objective of this analysis was inference rather than prediction, we provided a measure of predictive performance by calculating the mean area under the curve (AUC) of the receiver operating characteristic curve over stratified 10-fold cross-validation.^36^ Sensitivity analyses were conducted by rerunning the logistic regression models to include participants who did not know their HIV testing history (ever testing or testing in the past 12 months) as non-tested. Additionally, as fishing activities were predominantly reported by males, a descriptive subgroup analysis of the main outcomes was performed among male adults only.

### Patient and public involvement

HIV district focal people, community members, adult study participants and the Ministry of Health leaders were involved in the study design, analysis and interpretation of results.

## Results

### HIV prevalence and testing

**Figure 2:**
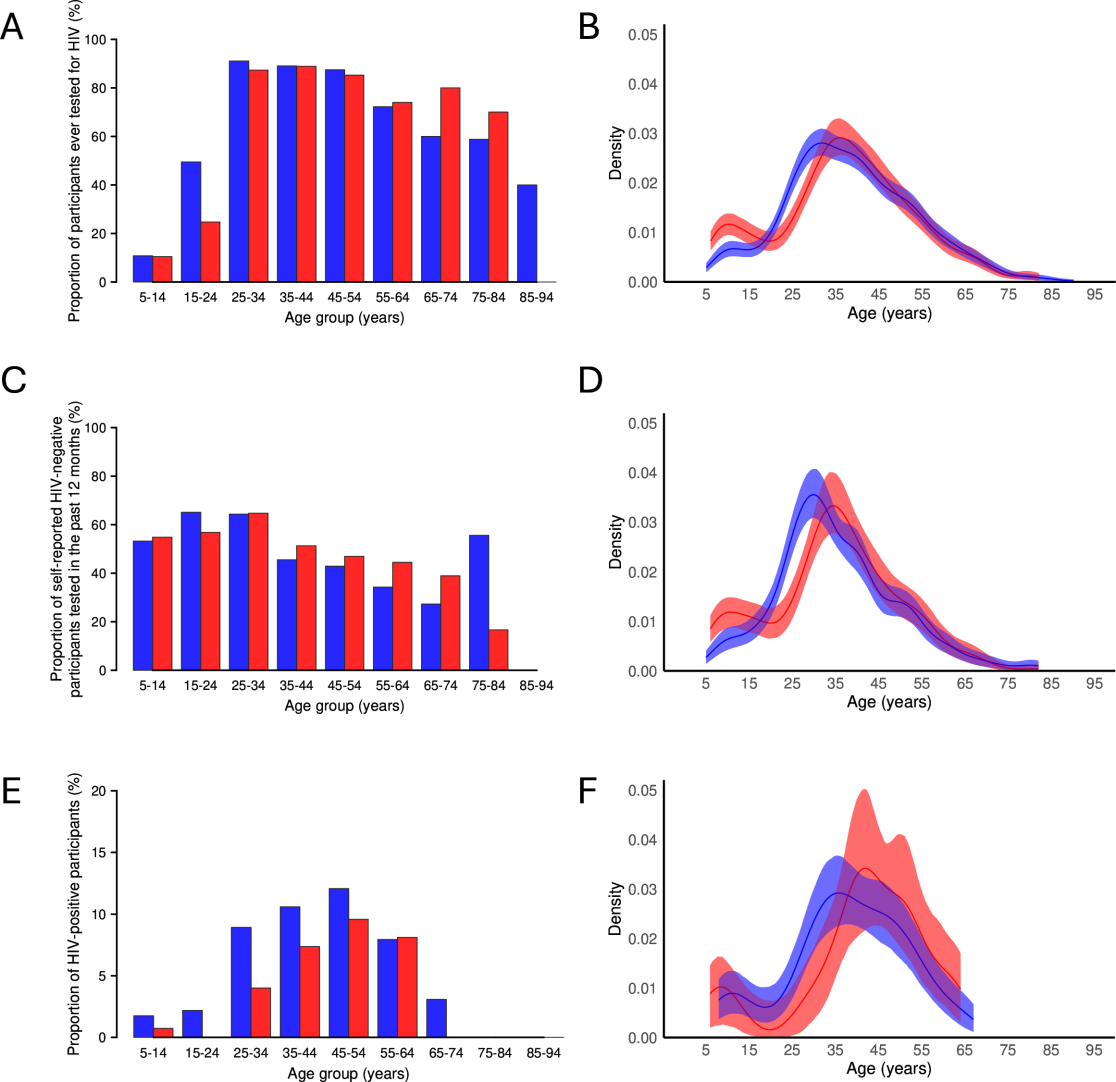
HIV testing coverage and prevalence by age and gender**.** (**A**) Proportion of participants ever tested for HIV (n=3197). (**B**) Kernel density plot of ever testing. (**C**) Proportion of self-reported HIV-negative participants tested for HIV in the past 12 months (n=1452). (**D**) Kernel density plot of testing in the past 12 months. (**E**) Proportion of HIV-positive participants (n=3197). (**F**) Kernel density plot of HIV positivity. Males—red, females—blue.

A total of 3395 participants aged 5 years and older from 1913 households were examined in 2024 (figure 1). 188 participants did not have HIV testing or history data available, leaving 3197 clinical participants from 1873 households. 124 participants who tested positive for HIV by Determine were tested for viral load in 2025. Study participant characteristics and HIV prevalence by subgroups are summarised in table 1 and online supplemental tables S2–S5. HIV prevalence in the entire SchistoTrack cohort was 4.66% (149/3197), including 1.19% (15/1261) in children younger than 15 years. Proportion and kernel density plots of ever testing, testing recency and HIV positivity, stratified by age and gender, are shown in figure 2.

**Table 1:** Adult participant characteristics (aged 15 years and older), stratified by ever testing for HIV and HIV infection status

	Overall (n=1931)	Non-HIV tested (n=471)	HIV tested (n=1460)	HIV-negative (n=1797)	HIV-positive (n=134)
Sociodemographic					
Fishing activities	415 (21.5%)	66 (14.0%)	349 (23.9%)***	390 (21.7%)	25 (18.7%)
Fishmongering activities	155 (8.0%)	24 (5.1%)	131 (9.0%)**	137 (7.6%)	18 (13.4%)*
Age	37 (27–49)	25 (16–50)	39 (31–49)***	37 (27–49)	41 (34–51)***
Gender					
Male	750 (38.8%)	225 (47.8%)	525 (36.0%)***	713 (39.7%)	37 (27.6%)**
Female	1181 (61.2%)	246 (52.2%)	935 (64.0%)	1084 (60.3%)	97 (72.4%)
Education					
None	387 (20.0%)	79 (16.8%)	308 (21.1%)***	362 (20.1%)	25 (18.7%)
Primary	1290 (66.8%)	356 (75.6%)	934 (64.0%)	1195 (66.5%)	95 (70.9%)
Secondary or above	254 (13.2%)	36 (7.6%)	218 (14.9%)	240 (13.4%)	14 (10.4%)
Occupation					
None or other	1048 (54.3%)	317 (67.3%)	731 (50.1%)***	975 (54.3%)	73 (54.5%)
Fisherman	230 (11.9%)	32 (6.8%)	198 (13.6%)	213 (11.9%)	17 (12.7%)
Fishmonger	113 (5.9%)	18 (3.8%)	95 (6.5%)	103 (5.7%)	10 (7.5%)
Subsistence farmer	530 (27.4%)	103 (21.9%)	427 (29.2%)	497 (27.7%)	33 (24.6%)
Rice farmer	10 (0.5%)	1 (0.2%)	9 (0.6%)	9 (0.5%)	1 (0.7%)
Majority tribe	1471 (76.2%)	353 (74.9%)	1118 (76.6%)	1377 (76.6%)	94 (70.1%)
Majority religion	1459 (75.6%)	354 (75.2%)	1105 (75.7%)	1358 (75.6%)	101 (75.4%)
Health behaviour					
Alcohol status	305 (15.8%)	44 (9.3%)	261 (17.9%)***	281 (15.6%)	24 (17.9%)
Smoking status	203 (10.5%)	31 (6.6%)	172 (11.8%)**	188 (10.5%)	15 (11.2%)
Household-level					
Main type of health facility used					
Private clinic or other	290 (15.0%)	81 (17.2%)	209 (14.3%)	269 (15.0%)	21 (15.7%)
Govt health centre	1641 (85.0%)	390 (82.8%)	1251 (85.7%)	1528 (85.0%)	113 (84.3%)
Social status	255 (13.2%)	62 (13.2%)	193 (13.2%)	236 (13.1%)	19 (14.2%)
Home owned	1689 (87.5%)	417 (88.5%)	1272 (87.1%)	1575 (87.6%)	114 (85.1%)
Home quality score	3 (3–9)	3 (3–9)	3 (3–9)	3 (3–9)	3 (3–9)
No of individuals in a household	3 (3–4)	3 (3–4)	3 (3–4)	3 (3–4)	4 (3–4)
No of years lived in a village	18 (8–30)	20 (10–35)	17 (8–30)***	19 (8–30)	15 (6.3–27)*
Year of recruitment					
2024	230 (11.9%)	31 (6.6%)	199 (13.6%)***	212 (11.8%)	18 (13.4%)
2023	440 (22.8%)	104 (22.1%)	336 (23.0%)	408 (22.7%)	32 (23.9%)
2022	1261 (65.3%)	336 (71.3%)	925 (63.4%)	1177 (65.5%)	84 (62.7%)
Spatial					
Distance to the nearest govt health centre (km)	2.6 (1.5–4.8)	2.7 (1.4–4.8)	2.6 (1.6–4.8)	2.7 (1.6–4.8)	2.3 (1.5–4.1)*
District					
Mayuge	462 (23.9%)	119 (25.3%)	343 (23.5%)	435 (24.2%)	27 (20.1%)
Buliisa	632 (32.7%)	149 (31.6%)	483 (33.1%)	585 (32.6%)	47 (35.1%)
Pakwach	837 (43.3%)	203 (43.1%)	634 (43.4%)	777 (43.2%)	60 (44.8%)

Data are presented as n (%) or median (IQR). The t-tests were performed for normally distributed numerical variables, and Wilcoxon rank sum tests were used for (non-normally distributed) numerical variables. χ2 tests were performed for categorical variables, except for occupation, where Fisher’s exact test was applied due to small counts in some categories. *p<0.05, **p<0.01, ***p<0.001.

HIV prevalence among adults aged 15 years and older was 6.94% (134/1931), with an age-standardised prevalence of 5.58%. 21.49% (415/1931) of participants reported fishing activities, among whom HIV prevalence was 6.02% (25/415) and age-standardised prevalence was 3.93%. By district, HIV prevalence was 5.84% (27/462) in Mayuge, 7.17% (60/837) in Pakwach and 7.44% (47/632) in Buliisa (χ² = 1.17, p=0.56). HIV prevalence in females was 8.21% (97/1181) and 4.93% (37/750) in males (χ² = 7.14, p=0.01).

Over 75% (1460/1931) of adults reported previously undertaking an HIV test. There were no significant differences between districts for individuals ever tested for HIV (χ² = 0.70, p=0.70). Nearly 69% (1331/1931) of participants reported never receiving a positive HIV test result. Of those, 3.68% (49/1331) did not know when they last tested for HIV, with the majority (85.71%, 42/49) being from Pakwach. Approximately half of self-reported HIV-negative participants who recalled when they last tested for HIV had been tested in the past 12 months (51.87%, 665/1282). Similarly, about half of those reporting fishing activities had tested in the past 12 months (52.23%, 164/314), with no significant difference between the groups (χ² = 0.01, p=0.94). 65.44% (284/434) of participants in Buliisa were tested for HIV in the past 12 months, compared with only 49.03% (152/310) in Mayuge and 42.57% (229/538) in Pakwach (χ² = 51.66, p<0.001). 51.29% (418/815) of females and 52.89% (247/467) of males were tested in the past 12 months (χ² = 0.24, p=0.62). Detailed data on HIV testing recency stratified by fishing activities are provided in online supplemental figure S2.

22.39% (30/134) of PWH were newly diagnosed within the SchistoTrack study, of whom 16.67% (5/30) reported fishing activities. Only four newly diagnosed individuals reported never having tested for HIV prior to the study. Among status-aware adult PWH, 5.77% (6/104) had been first diagnosed within the previous 12 months (none of whom reported fishing activities), 47.12% (49/104) 1–9 years ago, and 47.12% (49/104) 10 or more years ago (online supplemental figure S2). Most status-aware PWH had been diagnosed at a health clinic or hospital (93.27%, 97/104); only 4.81% (5/104) had been diagnosed through outreach mobile testing, and only one person reported being diagnosed at an HIV-specific testing and counselling centre. More status-aware PWH from Mayuge (20%, 4/20) had been diagnosed through outreach mobile testing than from Buliisa (0%, 0/38) and Pakwach (2.17%, 1/46) (Fisher’s p=0.005). Detailed information on testing locations stratified by fishing activities is shown in online supplemental figure S3.

### HIV care cascade

Among adult PWH reporting fishing activities, 80% (95% CI 64.32% to 95.68%, 20/25) were aware of their HIV status, and 76% (95% CI 59.26% to 92.74%, 19/25) were currently on ART. Self-reported viral suppression in this group was 100% (8/8) among PWH who had previously tested for viral load and knew the result. However, only 44% (11/25) of PWH reporting fishing activities recalled previously testing for viral load, and 32% (8/25) knew the result. Overall, 72% (95% CI 54.40% to 89.60%, 18/25) of all PWH reporting fishing activities were linked to care, 80% (95% CI 64.32% to 95.68%, 20/25) had initiated ART and 76% (95% CI 59.26% to 92.74%, 19/25) were retained in care (online supplemental figure 4). There were no significant differences in HIV care engagement between PWH reporting fishing activities and the general population or between districts (online supplemental figure 5). The results remained non-significant when the care cascade was re-examined using alternative fisherfolk definitions, including occupation (fishing or fishmongering) and fishmongering activities.

Overall, 88.06% (118/134) of adult PWH reported the HIV clinic where they accessed ART. Geographic coordinates were available for 86.96% (20/23) of the reported HIV clinics and 85.82% (115/134) of PWH. The median distance travelled to the reported HIV clinic was 6.73 km (IQR 2.64–11.61), compared with a median distance of 5.44 km (IQR 2.28–7.58) to the nearest HIV clinic (Wilcoxon W=561, p<0.001). Participants from Pakwach travelled shorter distances to their reported HIV clinics (median 2.45 km, IQR 1.76–5.88) than those from Buliisa (6.95 km, IQR 5.37–13.46) and Mayuge (11.61 km, IQR 11.00–12.45) (Kruskal-Wallis χ² = 44.86, p<0.001). There were no significant differences by fishing activities (Kruskal-Wallis χ² = 1.74, p=0.19). 71.30% (82/115) of PWH accessed ART at a clinic other than their closest facility, with no significant differences by fishing activities (χ² = 7.43 × 10^−31^, p=1.00). However, there were district differences: 89.13% (41/46) of PWH from Pakwach used their nearest clinic, compared with 57.69% (15/26) from Mayuge and 60.47% (26/43) of PWH from Buliisa (χ² = 11.97, p=0.003). Those who did not access ART at their nearest clinic travelled a median distance of 10.59 km (IQR 4.07–34.99) farther than necessary (ie, beyond the distance of the nearest clinic). Of those, PWH from Mayuge travelled a median additional distance of 1.18 km (IQR 0.79–10.33), compared with 34.26 km (IQR 6.86–39.90) in Buliisa and 21.03 km (IQR 12.58–34.94) in Pakwach (Kruskal-Wallis χ² = 12.09, p=0.002). Overall, 17.39% (20/115) of PWH received care outside their home district, with no significant differences by fishing activities or district (Fisher’s p=0.24 and p=0.06, respectively).

Among adult PWH aged 15 years and older, 76.12% (102/134), including 68% (17/25) of PWH reporting fishing activities, had their viral load measured in 2025 (figure 1). Among adult PWH with viral load, the median viral load was <839 copies/mL (limit of detection) (IQR <839–1124). 51.96% (53/102) of PWH had viral load below the limit of detection, while the viral load among those with detectable levels ranged from <839 to 2 03 423 copies/mL. 70.59% (72/102) of PWH were virally suppressed (<1000 copies/mL). Among unsuppressed PWH, 30% (9/30) had a low-level unsuppressed viral load between 1000 and 2000 copies/mL. Viral suppression among PWH reporting fishing activities (70.59%, 12/17) was comparable to that of the general population (70.59%, 60/85), with no significant differences between districts: 81.82% (18/22) in Mayuge, 67.65% (23/34) in Buliisa and 67.39% (31/46) in Pakwach. Among those newly diagnosed in 2024, the median viral load was <839 copies/mL (IQR <839–2980), with 61.90% (13/21) virally suppressed a year later. Of those unsuppressed, 25% (2/8) had a viral load between 1000 and 2000 copies/mL. Over half of PWH with measured viral load (64.71%, 66/102) reported previously testing for viral load, and roughly half (48.04%, 49/102) knew the result. Agreement between self-reported viral load and measured viral suppression a year later was 79.59% (95% CI 65.66% to 89.76%), with discordance driven by individuals reporting viral suppression when they were not actually suppressed (sensitivity 100% (95% CI 90.51% to 100%); specificity 16.67% (95% CI 2.09% to 48.41%)).

### Associations between fishing activities and HIV status and testing

Of the six fisherfolk definitions tested, community-level definitions, including the presence of a landing site in a village and household distance to the nearest water site, were uninformative for the main outcomes of ever testing, testing in the past 12 months and HIV status (table 2). The presence of a beach in the village was positively associated with HIV status and recent testing in unadjusted and minimally adjusted models, but was not selected in the BIC-based model. Fishmongering activities and occupation were crudely associated with ever testing, while only fishmongering activities were crudely associated with HIV status, but no fishmongering variables remained significant after adjustment for gender. The fishing occupation was associated with ever testing in unadjusted and minimally adjusted models, but not selected in the BIC-based model. Only fishing activities remained robust for ever testing across unadjusted, minimally adjusted and BIC-based models.

**Table 2:** Association of fisherfolk definitions with HIV testing and status

Outcome	Predictor	Unadjusted OR (95% CI)	Adjusted OR (95% CI)
Ever testing for HIV	Fishing activities	1.93 (1.46 to 2.59)***	3.40 (2.45 to 4.77)***
	Fishmongering activities	1.84 (1.19 to 2.94)**	1.38 (0.88 to 2.25)
	Occupation (reference: none or other) Fisherman Fishmonger	2.68 (1.83 to 4.05)*** 2.29 (1.39 to 3.97)**	3.53 (2.33 to 5.47)*** 1.65 (0.98 to 2.91)
	Landing site	0.89 (0.68 to 1.16)	0.87 (0.66 to 1.14)
	Beach	1.14 (0.93 to 1.41)	1.21 (0.92 to 1.59)
	Household distance to water site (m)	1.00 (1.00 to 1.00)	1.00 (1.00 to 1.00)
Testing in the past 12 months	Fishing activities	1.02 (0.79 to 1.32)	0.86 (0.61 to 1.22)
	Fishmongering activities	1.10 (0.75 to 1.64)	1.02 (0.67 to 1.58)
	Occupation (reference: none or other) Fisherman Fishmonger	0.94 (0.67 to 1.32) 1.27 (0.80 to 2.04)	0.86 (0.58 to 1.28) 1.05 (0.64 to 1.73)
	Landing site	0.96 (0.73 to 1.27)	0.88 (0.65 to 1.17)
	Beach	1.91 (1.53 to 2.39)***	1.44 (1.08 to 1.93)*
	Household distance to water site (m)	1.00 (1.00 to 1.00)	1.00 (1.00 to 1.00)
HIV infection status	Fishing activities	0.83 (0.52 to 1.27)	1.25 (0.70 to 2.22)
	Fishmongering activities	1.88 (1.08 to 3.11)*	1.49 (0.83 to 2.55)
	Occupation (reference: none or other) Fisherman Fishmonger	1.07 (0.60 to 1.80) 1.30 (0.61 to 2.48)	1.75 (0.89 to 3.40) 1.07 (0.50 to 2.09)
	Landing site	0.89 (0.59 to 1.38)	0.87 (0.57 to 1.36)
	Beach	1.54 (1.07 to 2.23)*	2.02 (1.28 to 3.23)**
	Household distance to nearest water site (m)	1.00 (1.00 to 1.00)	1.00 (1.00 to 1.00)

Evaluation of six fisherfolk definitions using unadjusted and minimally adjusted (for age, gender and district) logistic regression models. The main outcomes were ever testing for HIV, testing in the past 12 months and HIV status. Analyses were conducted among all study participants (n=1931) and among self-reported HIV-negative participants for the testing recency outcome (n=1282). Only the fisherman and fishmonger categories are shown for the occupation variable, which also includes subsistence farmers and rice farmers.

*p<0.05, **p<0.01, ***p<0.001.

**Figure 3:**
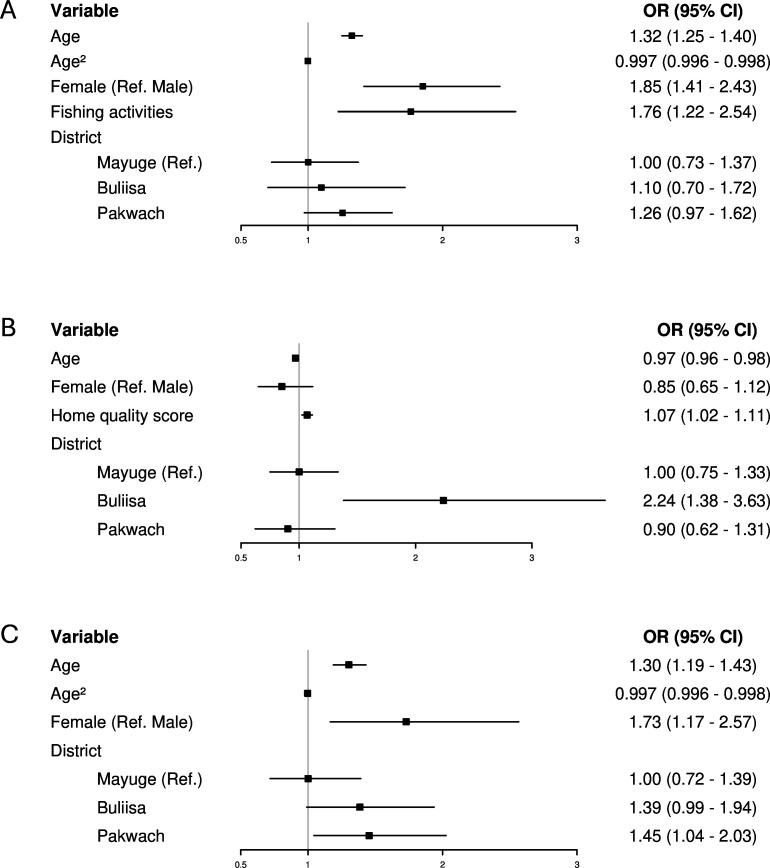
HIV testing and status models. (**A**) Model of ever testing for HIV (n=1931). (**B**) Model of testing for HIV in the past 12 months (n=1282). (**C**) Model of HIV status (n=1931). Logistic regression models were selected by backward stepwise selection based on the lowest Bayesian information criterion. 95% CIs were calculated using village-level clustered standard errors (number of village clusters=52). Floating absolute risks were calculated for the district variable. Intraclass correlation coefficient=0.041 (ever testing); 0.23 (testing recency) and 0.024 (HIV infection). Variance inflation factor range: 1.07–25.41 (ever testing); 1.03–1.43 (testing recency) and 1.02–34.43 (HIV infection). Area under the curve for stratified 10-fold cross-validation=0.75 (ever testing); 0.66 (testing recency); 0.67 (HIV infection).

The BIC-based model of ever testing for HIV included age, age^2^, gender, fishing activities and district (figure 3A). Older age was associated with higher odds of ever testing for HIV from 15 to 30 years of age (OR 1.32 (95% CI 1.25 to 1.40)). Beyond this age range, the effect of age was not relevant. Including the quadratic term for age resulted in an attenuated OR compared with the minimally adjusted model. Only 58.14% (357/614) of participants aged 15–30 had ever tested for HIV, compared with 83.75% (1103/1317) of participants aged over 30 years old. Female participants had 1.85 times higher odds of ever testing for HIV compared with male participants (95% CI 1.41 to 2.43). The proportion of ever tested participants was 79.17% (935/1181) in females and 70% (525/750) in males (χ² = 20.42, p<0.001). Participants reporting fishing activities had 1.76 times higher odds of ever testing compared with the general population (95% CI 1.22 to 2.54). 84.10% (349/415) of those reporting fishing activities and 73.28% (1111/1516) of the general population were ever tested for HIV (χ² = 20.07, p*<*0.001). Fishing activities were highly gendered: 91.33% (379/415) of participants reporting fishing activities were male. There were no consistent district effects across the models. The results remained robust after including participants who did not know their HIV testing history as non-tested and when analyses were restricted to male participants only (online supplemental figure S6 and table S7).

## Discussion

Fishing communities in Uganda have long been recognised as a priority population for the HIV response, yet they remain under-represented in national HIV surveillance data.^9^ We screened 3197 individuals aged 5–92 years for HIV in the SchistoTrack cohort in rural Eastern and Western Uganda. We analysed HIV prevalence, testing coverage and progress along the HIV care cascade in 1931 adults comprising the highest burden age group (aged 15 years and older). Our findings indicate that, in contrast to precedents, HIV prevalence in participants reporting fishing activities was not significantly higher than that of the general rural population. Participants who reported fishing activities reported higher lifetime HIV testing coverage and comparable care cascade engagement, although they still fell short of current UNAIDS targets.

The age-standardised HIV prevalence in SchistoTrack (5.6%) closely mirrors the national prevalence reported by UPHIA 2020–2021 (5.8%).^29^ The moderately higher crude prevalence (6.8%) reflects the older age distribution of the SchistoTrack cohort compared with UPHIA. Over one-fifth (22.4%) of PWH in our cohort were newly diagnosed. Nearly three-quarters (73.3%) of previously undiagnosed HIV cases were among women aged 25–52, which contrasts with broader trends across SSA, where women generally have better access to and uptake of HIV testing and care.^37^ However, this may reflect the higher overall HIV burden among women or potential gender biases in self-reported data.^1 38^

In our study, participants reporting fishing activities did not have higher HIV prevalence than the general population. Contrary to earlier HIV prevalence estimates in Ugandan fishing communities between 15% and 40% (8–13 years prior to our study), we found a much lower age-standardised prevalence of 3.9% and crude prevalence of 6.0% in participants reporting fishing activities.^7^,^9^ This may reflect our study locations being around the River Nile and Lake Albert, as opposed to Lake Victoria, where most previous studies of Ugandan fishing communities have taken place and where HIV prevalence is high.^8 11 12 19 20 39^ Additionally, differences in sampling methodology (recruitment from landing sites), exclusion of older adults and lack of age standardisation in other studies limit their generalisability and comparability.^10^,^1219 20^ Being fisherfolk was not relevant for identifying PWH at the individual level. Importantly, the lack of association between fishing activities and HIV status may also reflect the gendered nature of fishing: 91.3% of those reporting fishing activities were male, while the majority of PWH in our study were female. This gender imbalance likely lowers the measured prevalence among those reporting fishing activities and may obscure an underlying association with HIV risk. Possible gender differences in self-report might have further obscured the association of fishing activities with HIV status. While the presence of a beach in the village was associated with increased odds of HIV in unadjusted and minimally adjusted models, this was not retained after variable selection, suggesting that the community-level relevance of fisherfolk definitions needs additional exploration in studies with more diverse communities than those studied here. The suggested importance of distinguishing between individual and community-level definitions of fisherfolk also warrants further investigation to accurately define priority groups and efficiently use limited ART resources. There were no significant differences in HIV prevalence between districts, and prevalence was comparable to regional UPHIA estimates.

78% of adult PWH were already aware of their status, of whom 97% reported being on ART, and among those who knew their viral load result, 93% reported viral suppression. This is similar to the 81–96–92 cascade reported by UPHIA, where status awareness represents a key challenge to achieving the UNAIDS 95–95–95 targets.^29^ Among PWH reporting fishing activities, 80% were aware of their status, of whom 95% reported being on ART, and among those who knew their viral load result, all self-reported viral suppression. Despite this seemingly encouraging comparison, these results should be interpreted with caution, since many participants had either not tested for viral load due to being status-unaware or did not know their viral load result. If missing data were not an issue, viral suppression would likely be lower due to low status awareness and adherence issues. The care cascade among PWH reporting fishing activities was close to that observed by Burgos-Soto *et al*^10^ in fishing communities around Lakes Edward and George (86–99–87). As in that study, care engagement among PWH reporting fishing activities was comparable to that of the general rural population, contributing to evidence that challenges expectations of poorer outcomes in this priority population. Yet these findings potentially indicate the success of targeted HIV care engagement interventions among fisherfolk in Uganda, who have historically been defined as a priority population.^8 9^

Measured viral suppression, available for a subset of participants who tested HIV-positive a year previously, was comparable between all adult PWH and those reporting fishing activities (70.6%), but was considerably lower than the national target of 85.7% among all PWH.^17^ Only 61.9% of newly diagnosed adult PWH were virally suppressed 1 year after diagnosis, despite being linked to care and initiating ART. While 30% of those unsuppressed had relatively low viral load (1000–2000 copies/mL), suggesting possible measurement error, eight PWH had high viral loads (>10 000 copies/mL) despite ART enrolment. Poor ART adherence (rather than treatment resistance) was the main challenge in this cohort, based on poststudy follow-up of unsuppressed participants by the district HIV counsellors. The agreement between self-reported and measured viral load a year later was 79.6%, demonstrating that self-reported data can serve as a reliable proxy for viral suppression in the absence of laboratory results. This agreement rose to 81.6% when the low but unsuppressed individuals were considered as suppressed. Notably, discordance was mainly driven by participants who self-reported viral suppression but were not suppressed in the following year. Future studies should examine whether this reflects misconceptions about viral suppression, actual treatment failure or unreported adherence issues. Moreover, examining ART regimens through health records and assessing potential adherence challenges could help contextualise viral load findings. Many PWH (71.3%) in our study accessed ART at clinics farther from their nearest facility. Additional research is needed to understand whether individuals chose to travel longer distances due to concerns about stigma, heterogeneous healthcare infrastructure or because they selected services closer to their workplace or fishing locations. If this behaviour reflects an active individual choice, it underscores the need for more accessible service delivery that accommodates personal preferences and needs.

Although most HIV outcomes analysed did not differ between those reporting fishing activities and the general population, these communities fell short of UNAIDS targets for care of PWH. Additionally, 24% of SchistoTrack participants had never tested for HIV, and 48% had not tested in over a year, estimates comparable to UPHIA reports (22% and 57%, respectively).^29^ Individual-level definitions of fisherfolk were more relevant for ever testing than community-level definitions, further emphasising the need for more research into the importance of distinguishing between these levels. Fewer than 1% of previously tested individuals reported their last test or diagnosis was through HIV self-testing, revealing potential gaps in outreach efforts. Yet participants reporting fishing activities were more likely to have ever been tested for HIV, possibly reflecting prior targeted testing campaigns. Further work is needed to assess the apparently limited uptake of self-testing, given local knowledge (from district HIV counsellors) of past and current peer-based self-testing interventions in the study districts, and the literature indicating its suitability for reaching hard-to-reach, mobile fisherfolk.^18 40 41^ The choice of testing modality may also affect different stages of the care cascade in distinct ways. For example, a comparison of home-based and outreach event-based HIV testing in Ugandan fishing communities around Lake Victoria found that home-based testing identified more undiagnosed cases but was linked to lower rates of linkage to care compared with event-based testing.^42^

The main strengths of our study lie in its methodological approach. Using BIC-based models promotes parsimony by penalising complexity and reducing overfitting, in contrast to approaches such as Bayesian variable selection or an even more lenient measure such as likelihood ratio tests. Considering unadjusted, minimally adjusted and BIC-based models strengthened the robustness of the observed associations. We also systematically evaluated several individual and community-level fisherfolk definitions to determine their relevance for HIV outcomes. Our study also has some limitations. Care cascade estimates were based on prevalent rather than follow-up data and relied solely on self-reports, potentially introducing memory error, recall and social desirability bias. The observed 100% ART uptake among those aware of their status may have been due to misreporting if participants were aware of their status primarily because of their ART use. Moreover, the 12-month gap between self-reports and viral load measurement limits our ability to draw inferences on the full care cascade, particularly if there is bias in who among PWH returned for follow-up. In addition, the relatively high limit of detection for DBS (839 copies/mL compared with typical thresholds of 50 or 200), lenient viral suppression threshold and absence of cascade measure collection alongside the 2025 sample collection further limit interpretability. We also lacked detailed follow-up information on the exact ART regimen, individual adherence and repeated viral load measures, which will be important in future studies to understand differences between viral load measurement error, treatment failure and compliance issues. The relatively small number of PWH may have limited the statistical power to detect significant effects, potentially explaining the discrepancy between our findings and those of other studies reporting higher HIV prevalence (17.5–41.3%) that have demonstrated significant differences.^10^,^1220^ Importantly, this study should be replicated in other fishing community settings in Uganda and elsewhere in SSA to assess generalisability.

## Conclusion

Expanding community-based outreach, diversifying testing modalities and addressing structural and mobility-related barriers remain critical to improving HIV status awareness and ensuring linkage to care. This study demonstrates the feasibility of leveraging established population-based studies, such as SchistoTrack, to generate representative estimates of HIV burden, prevention and care cascade engagement in underserved populations. We showed that individuals reporting fishing activities in rural Uganda have higher lifetime HIV testing rates and comparable HIV prevalence and care cascade engagement to the general population. These findings suggest that prior targeted outreach efforts may have contributed to improved testing uptake among fisherfolk. However, significant gaps persist in recent testing, status awareness and viral suppression, underscoring the continued vulnerability of this priority population. To accelerate progress towards the UNAIDS 95–95–95 targets, it is crucial to continue to explore new strategies that promote regular HIV testing and sustained viral suppression.

## Supplementary material

10.1136/bmjopen-2025-108718 online supplemental file 1

10.1136/bmjopen-2025-108718 online supplemental file 2

## Data Availability

Participant data are not available due to the identifiable nature of the participant characteristics and the ongoing nature of the cohort. The analysis code is provided as online supplementary material.
