# Supplementary material for: HIV testing and prevalence in fishing communities in rural Uganda: a cross-sectional study of 3197 individuals within SchistoTrack
Source: BMJ Open. 2026 Feb 4;16(2):e108718. doi: 10.1136/bmjopen-2025-108718 (PMC12878462; doi:10.1136/bmjopen-2025-108718)
Supplement: Supplementary file 3 [file bmjopen-2025-108718.reviewer_comments.pdf]

PEER REVIEW HISTORY

BMJ Open publishes all reviews undertaken for accepted manuscripts. Reviewers are asked to complete a checklist review form and are provided with free text boxes to elaborate on their assessment. These free text comments are reproduced below.

ARTICLE DETAILS

Title (Provisional)

HIV testing and prevalence in fishing communities in rural Uganda: a cross-sectional study of 3197 individuals within SchistoTrack

Authors

Bui, Hanh Lan; Wilburn, Lauren; Nsimbe, Salim W; Nabatte, Betty; Oromcan, Geoffrey W; Mujuni, Raymond; Nabonge, Juma; Kabatereine, Narcis B; Smith, Adrian; Chami, Goylette

---

VERSION 1 - REVIEW

**Reviewer**	**1**
**Name**	**Apelian, Herak**
**Affiliation**	**Public Health Agency of Canada, STBBI Surveillance Division**
**Date**	**23-Oct-2025**
**COI**	**None**

This cross-sectional analysis used data from the SchistoTrack community-based cohort in rural Uganda to examine HIV prevalence, testing, and the care cascade among fisherfolk, compared to the general population. The authors also built univariate and multivariate logistic regression models with HIV-testing and HIV-status as outcomes, and assessed 6 definitions of fisherfolk. I commend all of this important work that the authors have done and I recommend its publication, with minor revisions. I invite the authors to consider the following comments.

Abstract -Introduction line 32: I suggest mentioning that Fisherfolk are recognized as a priority population for HIV control in Sub-Saharan Africa.

Abstract - Methods line 42: At the first mention of 1931 adult participants, I suggest adding that this is defined as those 15 years or older.

Introduction:

Line 108: It would be helpful to add the years to which to the estimated HIV prevalence of fisherfolk in Uganda (15-40%) refers, for more context, and to compare it to the current most recent analysis.

Methods page 4: I suggest adding some brief information on the SchistoTrack cohort: When did it start? What are the eligibility criteria to join the cohort? What was the recruitment method and how were participants recruited?

Methods page 4 line 179: add the DBS acronym after dried blood spot as it is used again in the discussion

Results and Table 2: Authors mention that no definitions of fisherfolk were associated with recent testing, however table 2 shows statistically significant ORs for Beach: 1.91 (1.53–2.39) 1.44 (1.08–1.93). The significance signs ** are also missing in Table 2.

Discussion page 10 line 409: spell out the first use of UPHIA

Page 10 line 419: Again, it would be good to know the year associated to the prevalence estimates between 15-40%

Page 11 line 443: This is a very important note added by the authors: to interpret the proportion of viral load suppression with caution, can the authors also comment on how they think the result would have been affected if these were not missing

Discussion limitations section page 12: No variable selection method to build multivariable adjusted regression models is perfect, however I suggest that the authors comment on their method of variable selection (BIC etc.) and how this is compared to other methods used. I also suggest that the authors highlight some of the strengths of their work.

Figures: I assume the figure titles and legends mentioned in page 15 will be coupled with their corresponding figures in pages 18 to 20?

**Reviewer**	**2**
**Name**	**Ha, Toan**
**Affiliation**	**University of Connecticut**
**Date**	**25-Nov-2025**
**COI**	**None**

This study provides valuable insights into HIV prevalence, testing, and care cascade engagement in rural Ugandan fishing communities, a priority population that is often underrepresented in research. The use of a large cohort and rigorous analytical methods, including age-standardization, gives this study meaningful value to existing literature. I have some comments below

General:

• Throughout the manuscript, please replace “participants were living with HIV (PLHIV)” with “participants with HIV (PWH)” to align with current inclusive language recommendations.

• Please clarify whether the study defines participants aged ≥15 years as “adult participants.” This should be explicitly stated, since legal definitions of adulthood vary. Any existing citation for this definition?

Abstract

• Clarify clarifying why fisherfolk are considered a priority population.

• State that the analyses of HIV prevalence, testing, and fisherfolk associations focus on adults aged 15 years and older. This prevents confusion since the full cohort includes participants aged 5–92 years.

• Spell out 6.02% at the beginning of the sentence.

Methods

• Ensure that the Methods describe both the full cohort (3197 participants aged 5–92 years) and the adult subset (1931 participants aged ≥15 years).

• The statement “Subsequent analyses focus on adults aged 15 years and older” should be moved to the Data Analysis section, and explicitly note that participants younger than 15 years were excluded from these analyses.

Results

• Spell out numbers at the beginning of sentences: For example, instead of “124 participants,” write “One hundred twenty-four participants” when starting a sentence (line 258).

Discussion

• The author reported that most participants reporting fishing activities are male, while most PLHIV are female. Pls discuss how this gender imbalance may influence observed prevalence and care cascade patterns. Because women generally have higher HIV prevalence in this study, and the fisherfolk group is mostly men, the measured HIV prevalence among fisherfolk may appear lower than expected. This could create the impression that fishing activities are not associated with higher HIV prevalence. Potential bias in self-report is also another issue.

VERSION 1 - AUTHOR RESPONSE

REVIEWER #1

Mr. Herak Apelian, Public Health Agency of Canada, Research Institute of the McGill University Health Centre

Reviewer 1, comment 1: This cross-sectional analysis used data from the SchistoTrack community-based cohort in rural Uganda to examine HIV prevalence, testing, and the care cascade among fisherfolk, compared to the general population. The authors also built univariate and multivariate logistic regression models with HIV-testing and HIV-status as outcomes, and assessed 6 definitions of fisherfolk. I commend all of this important work that the authors have done and I recommend its publication, with minor revisions. I invite the authors to consider the following comments.
Author response: Thank you for the supportive response and helpful feedback that has greatly improved the clarify of the manuscript.

Reviewer 1, comment 2: Abstract -Introduction line 32: I suggest mentioning that Fisherfolk are recognized as a priority population for HIV control in Sub-Saharan Africa.
Author response: We had to reformat the abstract to meet the journal guidelines so we no longer had an option to provide a background section and could not include this information.

Reviewer 1, comment 3: Abstract - Methods line 42: At the first mention of 1931 adult participants, I suggest adding that this is defined as those 15 years or older.
Author response: Apologies, but the abstract has now lost its Methods section due to the structure of BMJ Open abstracts. We do mention the definition though on the first use of the term 'adults' in the results.

Reviewer 1, comment 4: Introduction: Line 108: It would be helpful to add the years to which to the estimated HIV prevalence of fisherfolk in Uganda (15-40%) refers, for more context, and to compare it to the current most recent analysis.
Author response: Thank you for this comparison. We added the years of the studies (based on a systemic review) to indicate the studies were conducted between 2009-2016. We also highlight now in the discussion that the previously high prevalence observed in Ugandan fishing communities were from studies 8-13 years prior to our study.

Reviewer 1, comment 5: Methods page 4: I suggest adding some brief information on the SchistoTrack cohort: When did it start? What are the eligibility criteria to join the cohort? What was the recruitment method and how were participants recruited?
Author response: Thank you. We have added a sentence in the introduction about the purpose of the cohort. And, in the Methods, we expand to describe the recruitment strategy and start of the cohort as well as provide an updated citation to the cohort protocol paper.

Reviewer 1, comment 6: Methods page 4 line 179: add the DBS acronym after dried blood spot as it is used again in the discussion
Author response: Added.

Reviewer 1, comment 7: Results and Table 2: Authors mention that no definitions of fisherfolk were associated with recent testing, however table 2 shows statistically

significant ORs for Beach: 1.91 (1.53–2.39) 1.44 (1.08–1.93). The significance signs ** are also missing in Table 2.
Author response: Thank you for spotting this error. We have now added the significance signs, and checked all other variables have significance signs where needed. The in-text write up also has been corrected.

Reviewer 1, comment 8: Discussion page 10 line 409: spell out the first use of UPHIA Page 10 line 419:
Author response: Done.

Reviewer 1, comment 9: Again, it would be good to know the year associated to the prevalence estimates between 15-40%
Author response: Added as per comment above.

Reviewer 1, comment 10: Page 11 line 443: This is a very important note added by the authors: to interpret the proportion of viral load suppression with caution, can the authors also comment on how they think the result would have been affected if these were not missing.
Author response: Thank you for highlighting this needed addition. We have added the following sentence "If data missingness was not an issue, viral suppression would likely be lower due to low status awareness and adherence issues.".

Reviewer 1, comment 11: Discussion limitations section page 12: No variable selection method to build multivariable adjusted regression models is perfect, however I suggest that the authors comment on their method of variable selection (BIC etc.) and how this is compared to other methods used. I also suggest that the authors highlight some of the strengths of their work.
Author response: Thank you; we have added more information on the choice of BIC method in the Discussion where the main advantage is the handling of collinearity and producing parsimonious models. We also mention this strength in the abstract bullet points of strengths and limitations.

Reviewer 1, comment 12: Figures: I assume the figure titles and legends mentioned in page 15 will be coupled with their corresponding figures in pages 18 to 20?
Author response: Yes, we kept separate to allow for the journal to format the figure and legend separately.

REVIEWER #2:
Dr. Toan Ha, University of Connecticut

Reviewer 2, comment 1: Comments to the Author: This study provides valuable insights into HIV prevalence, testing, and care cascade engagement in rural Ugandan fishing communities, a priority population that is often underrepresented in research. The use of

a large cohort and rigorous analytical methods, including age-standardization, gives this study meaningful value to existing literature. I have some comments below
Author response: Thank you for providing helpful clarifications to strengthen the manuscript.

Reviewer 2, comment 2: General: • Throughout the manuscript, please replace "participants were living with HIV (PLHIV)" with "participants with HIV (PWH)" to align with current inclusive language recommendations.
Author response: Changed throughout.

Reviewer 2, comment 3: • Please clarify whether the study defines participants aged ≥15 years as "adult participants." This should be explicitly stated, since legal definitions of adulthood vary. Any existing citation for this definition?
Author response: We have added a line to indicate this definition in the covariates section, and provide a justification for the cut-off in the statistical methods. We do not have an existing citation for this definition, but instead used statistical information to define the concentration of our outcomes within this age group to focus our analysis. We also clarify in the methods "Only participants aged 15 years or older (referred to as adults for reporting purposes, even though this age group includes older adolescents)…"

Reviewer 2, comment 4: Abstract • Clarify clarifying why fisherfolk are considered a priority population.
Author response: We've now had to remove this sentence to meet BMJ Open abstract formatting requirements where there is no background section.

Reviewer 2, comment 5: • State that the analyses of HIV prevalence, testing, and fisherfolk associations focus on adults aged 15 years and older. This prevents confusion since the full cohort includes participants aged 5–92 years.
Author response: We have clearly stated this focus now in the abstract.

Reviewer 2, comment 6: • Spell out 6.02% at the beginning of the sentence.
Author response: Changed.

Reviewer 2, comment 7: Methods • Ensure that the Methods describe both the full cohort (3197 participants aged 5–92 years) and the adult subset (1931 participants aged ≥15 years).
Author response: We have clarified the denominators used in analyses in the Statistical analysis section of the methods.

Reviewer 2, comment 8: • The statement "Subsequent analyses focus on adults aged 15 years and older" should be moved to the Data Analysis section, and explicitly note that participants younger than 15 years were excluded from these analyses.
Author response: We have moved this to the methods and made it clear where those younger than 15 years of age were excluded from further analysis.

Reviewer 2, comment 9: Results • Spell out numbers at the beginning of sentences: For example, instead of "124 participants," write "One hundred twenty-four participants" when starting a sentence (line 258).
Author response: Corrected throughout the manuscript.

Reviewer 2, comment 10: Discussion • The author reported that most participants reporting fishing activities are male, while most PLHIV are female. Pls discuss how this gender imbalance may influence observed prevalence and care cascade patterns. Because women generally have higher HIV prevalence in this study, and the fisherfolk group is mostly men, the measured HIV prevalence among fisherfolk may appear lower than expected. This could create the impression that fishing activities are not associated with higher HIV prevalence. Potential bias in self-report is also another issue.
Author response: We have added a statement in this light to the Discussion.
"Importantly, the lack of association between fishing activities and HIV status may also reflect the gendered nature of fishing: 91.3% of those reporting fishing activities were male, while the majority of PWH in our study were female. This gender imbalance likely lowers the measured prevalence among those reporting fishing activities and may obscure an underlying association with HIV risk. Possible gender differences in self-report might have further obscured the association of fishing activities with HIV status."

VERSION 2 - REVIEW

**Reviewer**	**1**
**Name**	**Apelian, Herak**
**Affiliation Division**	**Public Health Agency of Canada, STBBI Surveillance**
**Date**	**05-Jan-2026**
**COI**	

I commend all the work put by the authors and the edits post review, no major comment.
